# Plant-Based Diets in Adolescence: Cardiometabolic Benefits, Developmental Risks, and the Role of Fortification

**DOI:** 10.3390/nu18183019

**Published:** 2026-09-16

**Authors:** Bianca Simionescu, Alina Mariela Murgu, Adriana Mihai, Vasile Valeriu Lupu, Elena Roxana Buzilă, Alice Grudnicki, Manuel Florin Roșu, Ancuța Lupu, Bogdan Novac, Emil Anton, Otilia Novac, Oana Raluca Temneanu

**Affiliations:** 1 Iuliu Hațieganu University of Medicine and Pharmacy, 400012 Cluj-Napoca, Romania; 2 Grigore T. Popa University of Medicine and Pharmacy, 700115 Iași, Romania; vizireanu.adriana@email.umfiasi.ro (A.M.); vasile.lupu@umfiasi.ro (V.V.L.); elena-roxana.buzila@umfiasi.ro (E.R.B.); alice.azoicai@umfiasi.ro (A.G.); florin.rosu@umfiasi.ro (M.F.R.); ancuta.ignat1@umfiasi.ro (A.L.); bogdan.novac@umfiasi.ro (B.N.); emil.anton@umfiasi.ro (E.A.); otilia.novac@umfiasi.ro (O.N.); temneanu.oana@umfiasi.ro (O.R.T.)

**Keywords:** adolescence, plant-based diets, vegetarian, vegan, cardiometabolic health, micronutrients, bone health, fortification

## Abstract

Adolescence is a critical window in which dietary patterns stabilise while nutrient requirements remain high to support growth, bone accrual, and neurodevelopment. Plant-based diets are increasingly adopted by young people, yet paediatric evidence indicates that they should not be treated as a single exposure, because diet quality substantially modifies both benefits and risks. Healthy plant-based patterns—rich in whole grains, legumes, fruits, vegetables, nuts, and unsaturated fats—are associated with more favourable blood pressure, glycaemia, lipids, adiposity, and insulin sensitivity in adolescents, whereas patterns built on refined grains, sugar-rich foods, and ultra-processed plant products are associated with the opposite profile. Recent paediatric meta-analyses identify recurrent vulnerabilities for vitamin B12, iron, zinc, calcium, and vitamin D, alongside a leaner phenotype that includes lower bone mineral content and, in vegans, shorter stature. The commercial food environment is a further determinant: audits of plant-based meat alternatives show that most are ultra-processed, that salt frequently exceeds national targets, and that only a minority are fortified. Much adolescent-specific evidence remains low-certainty and cross-sectional. The relevant question is therefore not whether plant-based diets are beneficial or harmful in adolescence, but under which conditions they improve cardiometabolic health without compromising growth, bone accrual, or micronutrient sufficiency.

## 1. Introduction

Plant-based diets have shifted from a niche dietary choice to a mainstream public health and sustainability topic, including within families with children and adolescents [1,2,3]. This transition is driven by concern for cardiometabolic disease burden, environmental impact, and food system sustainability [2,4].

Adolescence is a pivotal life stage for evaluating this transition, because cardiometabolic risk factors frequently emerge early, eating habits established during this period track into adulthood, and nutritional demands remain high [5,6]. Paediatric nutrition cannot, however, be extrapolated from adult evidence: growth velocity, pubertal development, and bone mineralisation impose safety constraints that are far less relevant in mature populations [1,2,7].

A key advance in understanding plant-based diets in youth involves recognising that diet quality fundamentally shapes outcomes. In one longitudinal trajectory analysis running from adolescence into young adulthood, healthy plant-based patterns tracked with favourable cardiometabolic profiles, particularly in females, whereas unhealthy patterns tracked with adverse metabolic outcomes, especially in males [5]. The magnitude of this quality distinction suggests that plant-based diet status alone—without attention to food composition—is an insufficient exposure metric. Food-group data show what this means in practice: vegan children and adolescents consumed more legumes, nuts, whole grains, milk alternatives, and meat alternatives than vegetarians, and less fat and sweet food, while omnivore intake of legumes and alternatives was close to zero [8].

A second problem is conceptual. The term “plant-based” frequently aggregates dietary patterns that differ sharply in nutrient density and cardiometabolic quality. Diets centred on legumes, intact grains, fruits, vegetables, nuts, and unsaturated fats are biologically distinct from patterns high in refined starches, sugar-sweetened foods, salt-rich meat analogues, and ultra-processed snacks [4,9,10,11]. This distinction is now operationalised through plant-based diet indices that separate overall, healthy, and unhealthy plant-based eating; these indices show stronger benefits for healthy patterns than for plant-based eating overall, in adolescent [5] and adult [12] samples alike, and in the index-development work spanning both [11].

The present review synthesises the evidence on healthy versus unhealthy plant-based diets in adolescence, addressing four linked questions: (i) whether healthy plant-based patterns improve cardiometabolic health; (ii) whether these diets generate developmental risks related to growth, bone accrual, and micronutrient adequacy; (iii) how the commercial food environment, and specifically ultra-processed plant-based products, modifies the exposure; and (iv) how fortification and supplementation modify the overall balance [7,13].

## 2. Materials and Methods

This work is a narrative review. Peer-reviewed literature was identified through structured searches of PubMed, conducted in August 2026, supported by an artificial-intelligence-assisted academic search engine (Consensus (Consensus NLP, Inc., San Francisco, CA, USA)) used to identify additional candidate literature; the tool did not itself determine inclusion, and all screening and final inclusion decisions were made by the authors against the criteria described below. Screening was performed independently by several authors, with disagreements resolved by discussion among the full author team. Every citation retained in this manuscript was subsequently verified against the indexed record of the source—authors, title, journal, year, volume, and pagination—and any reference that could not be located in PubMed or in the publisher’s own record was excluded, not retained. Search terms combined “plant-based diet”, “vegetarian”, “vegan”, “adolescents”, “children”, “cardiometabolic risk”, “micronutrient status”, “bone mineral density”, “bone mineral content”, “plant-based meat alternatives”, “ultra-processed foods”, “disordered eating”, “fortification”, and “supplementation”, combined using standard Boolean operators (AND/OR). As this is a narrative rather than a systematic review, no formal assessment of individual-study risk of bias was performed; certainty and risk-of-bias ratings reported elsewhere in this manuscript (e.g., Section 6 and Section 10.3) are reproduced from the source meta-analyses cited rather than independently assessed by the authors.

Priority was given to systematic reviews and meta-analyses, prospective cohort studies, validated dietary-index studies, randomised controlled trials, and society position papers concerning paediatric or adolescent populations published between 2018 and 2026. Cross-sectional market audits were included where they characterised the composition of commercially available plant-based products. Adult cohorts and meta-analyses were retained selectively when they provided mechanistic support or established biological plausibility for findings observed in younger populations. Because roughly one third of the evidence base in this field is adult-derived, we adopted an explicit labelling convention: every claim resting on adult data names that population at the point of use, and every claim resting on paediatric data below adolescence states the age range of the source. Market audits are identified as product-composition data, which describe what is on sale rather than what is eaten or what follows from eating it. Where a statement is our own inference rather than a reported finding, it is marked as such. Given the heterogeneity of dietary-pattern definitions and outcome measures across the included literature, findings were synthesised narratively and not pooled quantitatively.

## 3. Conceptualising Plant-Based Diet Quality in Adolescence

Plant-based diets exist along a continuum rather than as a single, uniform pattern [1,2,14]. Heterogeneity operates within the labels as well as between them. Principal component analysis of the German VeChi Youth Study identified three distinct dietary patterns among vegetarian children and adolescents and four among vegans, each with a different nutrient profile, so that more and less favourable patterns coexisted inside every diet group [15]. Exclusion category and diet quality are therefore separate axes, and the label alone locates an adolescent on only one of them. Vegetarian diets typically exclude meat and fish, whereas vegan diets exclude all animal-derived foods; plant-based indices widen the analytical lens by scoring the relative contribution of healthy versus less healthy plant foods, rather than the mere presence or absence of animal products [7,11,16].

This distinction is consequential because overall plant-based scores can obscure opposing biological signals. In adults, healthful plant-based patterns are associated with lower cardiometabolic risk, whereas unhealthful plant-based patterns tend to be associated with higher risk markers or poorer outcomes [17,18]. Adolescent and paediatric data now point in the same direction, although the evidence base remains considerably smaller [5,19,20]. A systematic review restricted to high-income settings reached a similar conclusion, identifying higher fibre intake and adequate energy from protein as benefits, and lower vitamin B12 and vitamin D intake as the principal risks [21].

## 4. Cardiometabolic Benefits of Healthy Plant-Based Diets

The strongest prospective adolescent evidence derives from the Planetary Health Diet Index (PHDI) study, which followed 886 Spanish adolescents over four years. High adherence (quartile 4 versus quartile 1) was associated with a markedly lower risk of new-onset high blood pressure, elevated plasma glucose, triglycerides, total cholesterol, and non-HDL cholesterol. Mixed models within the same cohort additionally linked higher adherence to lower glucose, lower triglycerides, and a lower BMI z-score over time [6]. Effect estimates of this magnitude—an 81% lower risk of new-onset high blood pressure—are considerably larger than those usually observed for dietary exposures, and warrant the caution appropriate to a single observational cohort of this size. The PHDI is a sustainability-oriented index rather than a plant-based diet index in the strict sense, so it should be read as a closely related but not interchangeable exposure.

A second longitudinal line of evidence comes from the Australian Raine Study, which tracked plant-based diet quality from adolescence into young adulthood in 417 participants. Diet quality remained relatively stable across life stages. Higher healthy plant-based trajectories in females were associated with lower insulin, lower HOMA-IR, lower systolic blood pressure, and lower hs-CRP, alongside higher HDL cholesterol; higher unhealthy plant-based trajectories in males were associated with larger waist circumference, greater waist-to-height ratio, higher insulin, higher HOMA-IR, and greater odds of hypertension [5]. This sex-specific divergence is discussed further in Section 10.1.

Cross-sectional adolescent obesity research supports the same quality split. Among 203 Iranian adolescents with overweight or obesity, the highest tertile of healthy plant-based diet adherence was associated with 84–85% lower odds of metabolically unhealthy obesity, whereas higher unhealthy plant-based adherence was associated with approximately fourfold higher odds; associations were stronger in girls, and in adolescents with overweight more than in those with obesity [19]. Comparable gradients have been reported in younger children: in Chinese children aged 6–9 years, higher healthful plant-based index scores were associated with higher lean mass and lower android fat mass percentage, and higher unhealthful scores with the reverse [22].

Lower LDL cholesterol is among the most consistently reported findings in the paediatric literature. A meta-analysis of 59 studies including 48,626 participants aged under 18 years—7280 lacto-ovo vegetarians, 1289 vegans, and 40,059 omnivores—reported lower total and LDL cholesterol in both lacto-ovo-vegetarian and vegan children [23]. A separate meta-analysis restricted to vegan children and adolescents aged 0 to 18 years, which included 18 studies, reported lower LDL alongside lower saturated fatty acid and cholesterol intakes, but also lower HDL cholesterol [24]. The lipid signal is therefore directional and not uniformly favourable: the atherogenic fraction falls, and so does the protective one. Whether the net effect on later cardiovascular risk is beneficial has not been established in any paediatric cohort.

Controlled evidence in this field comes almost entirely from adults. In an 8-week randomised trial in 22 pairs of identical twins (mean age 39.6 years), the vegan group showed significant reductions in LDL cholesterol, fasting insulin, and body weight relative to the omnivorous group [25]. The twin design supports causal inference for the lipid effect, but says nothing about growth, bone accrual, or pubertal development.

These findings are mechanistically coherent: healthy plant-based patterns are rich in fibre, micronutrients, and phytochemicals, and lower in saturated fat and energy density [2,10,26]. Adult mediation analyses suggest that lower adiposity, glycaemia, triglycerides, and inflammation explain part of the reduced type 2 diabetes risk associated with such patterns [27]. Adult cohorts and meta-analyses linking healthier plant-based patterns to lower cardiovascular and type 2 diabetes risk reinforce the biological plausibility of early-life prevention [12,16,18]. A synthesis of nine prospective adult cohorts found the same quality gradient for adiposity: overall plant-based scores were protective against weight gain, emphasising healthy plant foods strengthened the association, and emphasising unhealthy plant foods produced a positive or null one, with moderate certainty [28].

## 5. Developmental Risks: Growth, Bone, and Body Composition

The principal paediatric caution is that cardiometabolic improvement does not guarantee developmental adequacy. The most detailed phenotyping comes from a cohort of 5- to 10-year-old Polish children. There, vegan diets were associated with lower LDL cholesterol and lower hs-CRP, but also with lower vitamin B12, lower 25-hydroxyvitamin D in the absence of supplementation, lower iron status, higher homocysteine, shorter stature, and lower bone mineral content [7]. The same cohort produced a second result that complicates any simple reading. Lacto-ovo-vegetarian children had lower total and HDL cholesterol but higher glucose, VLDL, and triglycerides than omnivores, which the authors described as an unexpectedly less favourable cardiometabolic profile [7]. Within a single cohort, therefore, the two plant-based groups moved in opposite cardiometabolic directions—an observation that argues against exclusion status as an exposure metric and for the diet-quality framing adopted here. These data are not adolescent-specific, but they remain relevant because they expose a biological trade-off that plausibly persists into later developmental stages.

Meta-analytic evidence now supports this pattern across a much larger population. In the pooled analysis of 59 paediatric studies, lacto-ovo-vegetarian children showed lower height, weight, BMI z-scores, fat mass, and bone mineral content, while vegan children showed shorter stature and lower BMI z-scores; the authors characterised the overall result as a leaner phenotype and noted that group averages for most nutrients and biomarkers nonetheless remained within paediatric reference ranges [23]. The meta-analysis restricted to vegan children likewise found lower height compared with omnivorous peers, and rated the certainty of evidence as low or very low, with 7 of 18 included studies at high or very high risk of bias [24]. This qualification matters clinically: a lower mean height, weight, or bone mineral content at group level does not equate to individual growth impairment, and correct interpretation requires reference to pubertal stage, longitudinal growth trajectory, and age- and sex-specific paediatric reference ranges rather than to a single cross-sectional measurement.

Not all cohorts show this pattern. The German VeChi Youth Study of 149 vegetarian, 115 vegan, and 137 omnivore participants aged 6–18 years found lower ferritin in both plant-based groups, but no significant difference in haemoglobin, 25-hydroxyvitamin D, HDL cholesterol, or triglycerides, and concluded that it did not indicate specific nutritional risks [29].

### 5.1. Bone Accrual as the Critical Constraint

Bone outcomes are of particular importance, since adolescence is a period of rapid skeletal accrual. A healthy plant-based propensity score was associated with improved bone stiffness in the I.Family study, suggesting that skeletal outcomes may depend on dietary quality rather than on plant-based status per se [11]. Two caveats limit how far this can be taken: the analysis pooled children, adolescents, and adults, and the authors judged the scores valid and reliable in adults while explicitly noting difficulties in applying them to younger age groups.

The longitudinal Saskatchewan Paediatric Bone Mineral Accrual Study is consistent with this interpretation. Among 125 adolescents followed into young adulthood, a “vegetarian-style” dietary pattern independently predicted both adolescent and adult total-body bone mineral content. Participants in the third quartile had 8.5% higher young-adult total-body bone mineral content than those in the first. Dietary pattern scores tracked moderately from adolescence into adulthood [30]. An important caveat applies. The pattern was rich in dark green vegetables, non-refined grains, legumes, nuts, seeds, and fruit—but it also included eggs, 100% fruit juice, and low-fat milk. It is therefore plant-predominant rather than plant-exclusive. The contribution of dairy to the skeletal signal cannot be separated from that of the plant foods.

These findings should be read alongside evidence from long-term prospective cohorts showing that stricter exclusion carries skeletal consequences. EPIC-Oxford followed participants for an average of 17.6 years. After adjustment for socio-economic factors, lifestyle, and BMI, vegans had a higher risk of hip fracture than meat eaters (hazard ratio 2.31; 95% CI 1.66–3.22) and of total fracture (1.43; 1.20–1.70). The elevation in vegetarians was smaller (hip fracture 1.25; 1.04–1.50) [31]. Meta-analytic data in adults likewise indicate lower bone mineral density at the femoral neck and lumbar spine in vegetarians and vegans [32]. The fracture component of that analysis used crude, not adjusted, risk ratios and has been contested on methodological grounds. The mechanisms proposed for this excess risk—lower calcium and vitamin D intake, lower protein quantity and quality, and lower body mass index—are the same constraints identified in paediatric cohorts, which is why adult fracture data remain relevant even though they cannot be applied directly [33]. Bone-turnover markers support this reading: in Spanish adults, lacto-ovo vegetarians and especially vegans had lower protein, calcium, and vitamin D intakes, and vegans showed significantly higher parathyroid hormone and bone resorption markers than omnivores [34]. This is the adult counterpart of the parathyroid hormone trend described below in young children. These are nonetheless adult data; no equivalent fracture cohort exists in adolescents. A large cross-sectional study of young adult Slovak women (*n* = 648; 67 vegetarian) using radial and tibial quantitative ultrasound found higher speed-of-sound values, indicating better bone tissue quality, in vegetarians than in omnivores [35]—a finding that runs counter to the adult fracture and bone-turnover data above and underscores that skeletal outcomes in young adulthood may depend more on overall dietary adequacy than on vegetarian status per se.

A study of bone and mineral metabolism in 2- to 7-year-olds is informative here. Children following vegan or vegetarian diets showed a linear trend towards higher parathyroid hormone concentrations, despite adequate vitamin D status and approximately adequate calcium intake. Protein intake was lower in the plant-based groups (12–14% of energy) than in omnivores (16–17%) [36]. Protein intake and calcium bioavailability, rather than vitamin D alone, may therefore be limiting factors [36]. Consistent with this, reduced bone mineral content has been reported in vegetarian and, particularly, vegan children in the wider systematic literature [3]. Whether these differences reflect body size rather than bone quality remains contested [37]. In the Polish cohort the answer differed by diet: the deficit attenuated after accounting for body size in vegetarians, but persisted in vegans (total body minus head −3.7%, 95% CI −7.0 to −0.4; lumbar spine −5.6%, −10.6 to −0.5) [7].

### 5.2. Body Composition

Body composition findings point to a comparable quality gradient. The Generation R cohort assessed 3340 children at 8 years and followed them to 13 years. A higher healthful plant-based index score was associated with a higher fat-free mass index z-score (0.05 per 10-point increment; 95% CI 0.01–0.10) and a lower body fat percentage z-score (−0.05; 95% CI −0.09 to −0.01); a higher unhealthful score was associated with lower height, weight, and fat-free mass index (−0.08; 95% CI −0.12 to −0.03) [20]. Comparable gradients have been reported in cross-sectional paediatric samples [11,22]. This pattern is consistent with the broader observation that a plant-based diet may generate either a lean, metabolically healthy phenotype or a nutritionally constrained phenotype, depending on food quality and dietary adequacy rather than on dietary ideology [4,10,23]. Bioelectrical impedance analysis extends this picture beyond fat mass and BMI-based indices to cellular-level parameters: in a companion Slovak cohort of young adult women (*n* = 647; 66 vegetarian), vegetarians showed a significantly lower phase angle and a higher extracellular-to-total-body-water ratio than omnivores, differences that persisted after adjustment for smoking, physical activity, BMI, and waist-to-hip ratio [38]—a subtler and less reassuring signal than the anthropometric picture above, illustrating why body-composition assessment should not stop at fat mass and BMI.

## 6. Micronutrient Adequacy and the Limits of “Plant-Based” as a Safety Label

### 6.1. The Overall Nutrient Profile

Systematic review evidence indicates that nutrient vulnerabilities are not exclusive to plant-based diets, but that some are clearly amplified when animal-source foods are excluded. Across 30 paediatric studies, all dietary groups showed a risk of inadequate vitamin D and calcium intake, while paediatric plant-based diets were more likely to show inadequate vitamin B12, iron, and zinc [13]. This is clinically salient because these nutrients underpin erythropoiesis, skeletal mineralisation, immune competence, and neurological development.

The largest paediatric meta-analysis to date refines this picture. Lacto-ovo-vegetarian children consumed less energy, protein, fat, vitamin B12, vitamin D, and zinc, but more fibre, iron, folate, vitamin C, and magnesium; vegan children showed a similar profile with particularly low calcium intake. At the biomarker level, lower ferritin and 25-hydroxyvitamin D were observed in lacto-ovo vegetarians and lower haemoglobin and ferritin in vegans, with increased odds of iron deficiency and anaemia in lacto-ovo vegetarians and of vitamin B12 deficiency in vegans [23]. The vegan-specific meta-analysis reported lower protein, calcium, and riboflavin intakes and lower ferritin concentrations, but higher blood vitamin B12 concentrations attributable to supplement use [24].

Prospective data from the general paediatric population show that the same gradient operates below the threshold of vegetarianism. In the Generation R cohort, higher scores on all three plant-based diet indices were associated with lower intakes of vitamin B2, vitamin B12, and calcium; whereas, a higher healthful score was additionally associated with higher intakes of fibre, vitamin C, magnesium, and copper [20]. Moving towards a plant-predominant diet therefore improves some parts of the nutrient profile and degrades others simultaneously, and the two movements are not interchangeable: fibre and vitamin C are rarely limiting in children, whereas vitamin B12 and calcium can be.

The central methodological point of this section is that intake and status dissociate. The direction of a nutrient’s intake gradient does not reliably predict the direction of its biomarker gradient, because fortification, supplementation, and bioavailability intervene between the two. Vitamin B12 illustrates the dissociation in one direction—lower intake, higher blood concentrations, because supplement use is widespread [24]—and iron illustrates it in the other.

### 6.2. Iron: Higher Intake, Lower Status

Iron is the clearest example of a nutrient for which measuring intake is actively misleading. Paediatric meta-analytic data show that lacto-ovo-vegetarian children consume more iron than omnivores yet have lower ferritin and higher odds of iron deficiency and anaemia [23], and vegan children likewise show lower ferritin [24]. The same dissociation is documented in adults: a meta-analysis of 24 cross-sectional studies found serum ferritin 29.7 μg/L lower in vegetarians than in non-vegetarians, with a larger difference in men than in premenopausal women [39]. The explanation is bioavailability. Iron absorption has been estimated at 14–18% from mixed diets but only 5–12% from vegetarian diets in individuals with no iron stores [40]. These estimates derive from isotope studies in adults and underpin dietary reference values for the general population; no adolescent-specific absorption estimates for plant-based diets exist, so applying them to this age group is an extension rather than a direct measurement. Phytate, polyphenols, and calcium inhibit non-haem iron absorption; ascorbic acid and organic acids enhance it. In a varied diet containing multiple inhibitors and enhancers, the effect of any single component is more modest than single-meal isotope studies suggest [40].

Three factors converge in adolescence. Iron demand rises with expanding muscle mass, blood volume, and haemoglobin concentration. Menarche adds recurrent losses. And adolescents following vegetarian or vegan diets depend exclusively on non-haem iron, which has lower bioavailability and therefore requires a higher total intake [41]. Adolescents with disordered eating patterns, heavy menstrual bleeding, or high training loads sit at the intersection of several of these risks [41]. Iron deficiency in this age group is frequently unrecognised because it precedes anaemia by a considerable interval. It may also not be a purely haematological problem. In adults following omnivorous, lacto-ovo vegetarian, or vegan diets, iron deficiency was associated with elevated parathyroid hormone, low vitamin D status, and markers of bone loss [42]. The iron and skeletal vulnerabilities described in Section 5 and Section 6 may therefore be linked, not merely parallel.

Practical mitigation is well characterised, although the source recommendations were developed for vegetarian infants and young children and have not been formally trialled in plant-based adolescents. Recommended measures include fortified cereals; leavened in preference to unleavened whole grains; soaking dried legumes and discarding the soaking water; fermented soy foods and sprouted legumes; and replacing tea and coffee at meals with vitamin C-rich drinks, fruit, or vegetables [43]. These are food-preparation strategies rather than supplementation, and they are unusually well suited to adolescent counselling because they change how habitual foods are eaten without requiring new ones.

### 6.3. Zinc: A Genuine Evidence Gap

Zinc follows a similar bioavailability logic but with a substantially weaker evidence base. Zinc absorption from plant-based diets is reduced by the same phytate and polyphenol content that limits iron absorption, and lacto-ovo-vegetarian children have been shown to consume less zinc than omnivores [13,23]. However, serum zinc—the recommended population biomarker—has rarely been measured using the recommended collection and analytical procedures in this population, which makes the existing data difficult to interpret. What data exist show no difference in serum zinc or growth between young vegetarian and omnivorous children, alongside some evidence of low serum zinc concentrations in vegetarian adolescents specifically [43]. More recent adolescent-inclusive data are consistent with this. Among 342 participants aged 6–18 years in the VeChi Youth Study, zinc intake was lower in both vegans and vegetarians than in omnivores, and serum zinc was correspondingly lower in both groups. Selenium intake and both serum selenium and selenoprotein P were also lower, yet glutathione peroxidase-3 activity did not differ, indicating that functional selenium status was preserved [44]. The dissociation between a lowered biomarker and a preserved functional marker is precisely why serum concentrations alone should not be read as evidence of deficiency. Fortified foods reduce iron deficiency, but whether they also reduce zinc deficiency is less certain [43].

This is therefore a domain in which clinical caution and evidentiary honesty point in slightly different directions. The mechanistic case for reduced zinc bioavailability is strong; the evidence that this translates into measurable zinc deficiency in adolescents on plant-based diets in high-income settings is thin. We note it as a monitoring priority rather than as an established harm.

### 6.4. Vitamin B12 and Vitamin D

Vitamin B12 is the nutrient for which the evidence is least ambiguous and the remedy most straightforward. A meta-analysis of children and adolescents aged 5 to 18 years found significantly lower vitamin B12 concentrations in those following plant-based diets, with the effect concentrated in vegan and macrobiotic diets and absent in vegetarians; critically, total vitamin B12 intake nullified the difference [45]. Deficiency in this age group can cause irreversible neurological injury, which is why the risk warrants a categorical response, not a graded one.

Vitamin D intake is lower in both lacto-ovo-vegetarian and vegan children [23], but inadequate vitamin D intake is a feature of all paediatric dietary groups, not a plant-based-specific vulnerability [13]. This distinction matters for counselling: vitamin D is a population-level issue that plant-based diets aggravate; whereas, vitamin B12 is a vulnerability that plant-based diets create.

### 6.5. Iodine and Long-Chain Omega-3 Fatty Acids

Two further nutrients deserve explicit mention in adolescence. Iodine deficiency has been identified as a risk in both vegetarian and vegan children, with potential consequences for thyroid function [3]. The mechanism is largely a fortification failure rather than an intrinsic property of plant foods, and it has been quantified. A cross-sectional survey examined 300 plant-based dairy and fish alternatives in United Kingdom retail outlets. After excluding organic products, which cannot legally be fortified, only 28% of milk alternatives and 6% of yoghurt alternatives contained added iodine, against 88% and 73% respectively for calcium. No cheese alternative and no fish alternative was iodine fortified. Modelling substitution of three daily portions of dairy with unfortified alternatives gave a 97.9% reduction in iodine provided (124 versus 2.6 μg), reducing the contribution to the adult recommendation of 150 μg/day from 83% to 1.8% [46]. The fortification audit is product-level and therefore applies to any consumer, but the intake scenario used adult portions and adult reference values and has not been repeated with adolescent portions.

The asymmetry between calcium and iodine fortification is the clinically actionable point: the same product that responsibly replaces the calcium lost by excluding dairy usually does not replace the iodine. Because iodine requirements are elevated in pregnancy and thyroid hormones are critical to foetal neurodevelopment, this gap is of particular relevance for adolescent girls approaching menarche and reproductive age. In practice it means that “fortified plant milk” is not a sufficient specification in counselling; the label must be checked for iodine specifically.

Long-chain omega-3 fatty acid status and protein quality are similarly recognised as points of vulnerability. The 2025 ESPGHAN Nutrition Committee position paper identifies protein, omega-3 fatty acids, calcium, and iron as intakes requiring explicit attention in vegan children throughout the paediatric age range [47]. Quantitative paediatric data on omega-3 status in plant-based adolescents specifically remain sparse, and this is identified as a research gap in Section 10.4 and not as a basis for a specific supplementation recommendation.

Overall, the most consistently recurring deficiencies in stricter paediatric plant-based diets remain vitamin B12, iron and low ferritin, and vitamin D insufficiency, particularly among vegans [1,7,23]. This is why paediatric reviews and society position papers consistently emphasise that children and adolescents following vegan diets require structured nutritional supervision rather than generic reassurance [1,3,47].

## 7. The Commercial Food Environment: Plant-Based Meat Alternatives and Ultra-Processing

Any discussion of adolescent plant-based eating that ignores the commercial food environment is incomplete, because adolescents are heavy consumers of convenience foods and plant-based meat alternatives are marketed with an implicit health claim. Unusually for this field, the relevant evidence is abundant. Market audits have been conducted independently in several countries using comparable methods. This allows a question that single-country data cannot answer: is the observed pattern a local regulatory artefact, or a structural property of the product category?

### 7.1. What Market Audits Show

A cross-sectional survey compared 207 plant-based meat products with 226 meat products from 14 United Kingdom retailers. The plant-based products had lower energy density, total fat, saturated fat, and protein, and higher fibre, than their meat equivalents. Salt, however, was significantly higher in five of six plant-based categories, and nearly three-quarters did not meet the then-current United Kingdom salt targets. Applying the national nutrient profiling model, 14% of plant-based products versus 40% of meat products were classified as less healthy [48].

A German audit of 298 plant-based meat and sausage analogues against 294 animal-based reference products, collected in 2024 from ten retail chains representing over 70% of the national grocery market, reproduced this pattern in detail. Analogues contained less total fat, saturated fat, and protein but more fibre, carbohydrate, and sugar; Nutri-Scores were significantly more favourable in six of nine subcategories, driven mainly by lower saturated fat and higher fibre. Salt, however, was significantly higher in three of four meat-analogue categories, with the widest gap in red-meat analogues (1.8 versus 0.6 g/100 g) [49].

An Australian audit of 790 products found that meat analogues had a higher mean Health Star Rating and lower saturated fat and sodium, but higher total sugar, than meat products [50]. United States data show the same directional trade-off, with a starker sodium contrast. Nutrient density scores were significantly lower for meat and fish alternatives than for meat or fish; mean sodium was 660 mg/100 g against 60 mg/100 g for beef, and mean protein 17 g against 20 g per 100 g [51].

The pattern is therefore consistent across four independent markets: plant-based analogues are reliably better on saturated fat and fibre, reliably worse on protein quantity, worse or equivalent on salt depending on the comparator category, and variable on sugar. This is not a uniformly healthier product class; it is a product class with a different, and category-dependent, nutritional trade-off.

### 7.2. The Fortification Gap Is Internationally Consistent

The finding of greatest paediatric relevance concerns fortification, and here the international convergence is striking. In the Australian audit, only 12.1% of meat analogues were fortified with iron, vitamin B12, and zinc [50]. In the German audit, only 12% of products—37 of 298—were fortified with any micronutrient at all: 37 with vitamin B12, 36 with iron, three with zinc, and one with riboflavin. No product contained added vitamin D or iodine [49]. An earlier Australian audit of 137 products found somewhat higher but still minority rates: 24% fortified with vitamin B12, 20% with iron, and 18% with zinc. Only 4% were low in sodium [52]. Analysis of United States branded-product data reached the same conclusion qualitatively, reporting that fortification with the nutrients characteristically associated with animal-source proteins—B vitamins, vitamin D, iodine, zinc, and long-chain omega-3 fatty acids—was minimal [51].

The gap is not confined to meat analogues. A United Kingdom survey of 300 plant-based dairy and fish alternatives found that only 28% of milk alternatives were fortified with iodine, against 88% fortified with calcium. No cheese or fish alternative was iodine fortified at all [46]. This is the same selective pattern—fortifying some displaced nutrients but not others—in an adjacent product category.

Two markets on two continents, audited six years apart, converge on approximately one in eight meat-analogue products fortified. This consistency argues that the fortification gap is a structural feature of how these products are formulated rather than a consequence of any single country’s regulatory environment. Canada is reported to be an exception, having established minimum requirements for protein content and quality and for micronutrients including iron and vitamin B12 in meat analogues [49], which indicates that the gap is addressable by regulation.

A five-year follow-up survey of the same United Kingdom market allows the trend to be assessed directly, and the answer is mixed. Between 2020 and 2024 the proportion of iodine-fortified plant-based milk alternatives rose, but remained well below fortification rates for calcium and vitamin B12. Fortification varied by base: no rice-based drink contained iodine, whereas oat- and pea-based drinks were more often fortified. Iodine fortification remained nearly absent from cheese and yoghurt alternatives, no fortified seafood alternative was identified, and rates for calcium, riboflavin, and vitamin B12 did not change significantly over the period [53]. The gap is therefore narrowing for one product category and static for the rest.

The paediatric relevance follows directly from Section 6, although it remains inferential rather than measured: these audits describe what is offered for sale, not what adolescents actually purchase or consume. The micronutrients least often added to these products—vitamin B12, iron, zinc, vitamin D, and iodine—are precisely those identified as recurrently vulnerable in paediatric plant-based diets. The products most likely to displace meat in an adolescent diet are, in the great majority of cases, not fortified with the nutrients whose loss the exclusion of meat creates.

### 7.3. Ultra-Processing and What It Does and Does Not Imply

Plant-based meat analogues are routinely classified as ultra-processed under the NOVA system, and this is frequently treated as a self-evident indictment. Two observations complicate that reading.

First, the comparator is also ultra-processed. In the Australian audit, 84% of meat analogues were ultra-processed—but so were 89% of the conventional meat products against which they were compared [50]. Ultra-processing therefore does not distinguish the two categories; it characterises the processed-meat aisle as a whole.

Second, the additive burden is more modest than public discussion implies. In the German audit, meat analogues averaged roughly two additives per product and sausage analogues around three; methylcellulose was the single most common, appearing in 175 of 298 products, and 50 products contained no additives at all. Flavourings were present in 92% of products, usually without disclosure of type [49]. Wide variability within the category is the dominant finding, which supports counselling at the level of the product, not the category.

What can legitimately be said about health outcomes is more limited than either enthusiasts or critics usually allow. Higher ultra-processed food intake in general has been associated with overweight, obesity, and adverse cardiometabolic profiles in the majority of paediatric studies, although with heterogeneous methods and predominantly observational designs [54]. Evidence specific to this age group is accumulating. Among United States adolescents in NHANES 2007–2018, higher ultra-processed food intake was associated with poorer cardiovascular health [55], and a systematic review of ten studies found that longitudinal analyses with follow-up beyond four years reported positive associations with adiposity, whereas cross-sectional analyses did not [56]. However, no study has yet quantified ultra-processed plant-based food intake as a distinct exposure in adolescents. Extending the general ultra-processed food literature to plant-based analogues specifically is a reasonable inference, not an established finding.

### 7.4. From Product Composition to Health Outcome

The step from composition to outcome should not be taken for granted, and the one relevant trial suggests why. In an 8-week randomised controlled trial, 89 adults at elevated diabetes risk in Singapore substituted 2.5 daily servings of habitual protein-rich foods with either plant-based meat analogues or their corresponding animal-based meats. The primary outcome, LDL cholesterol, did not differ between groups, and no significant effect was observed on the wider lipid–lipoprotein profile. Diastolic blood pressure was lower in the analogue group, but glycaemic regulation was better in the animal-meat group, with interstitial glucose time in range of 94.1% versus 86.5%. The authors concluded that an 8-week plant-based meat analogue diet did not show widespread cardiometabolic benefit relative to a corresponding meat-based diet [57].

This trial was conducted in middle-aged adults with elevated metabolic risk and cannot be transferred to adolescents. Its methodological lesson nonetheless transfers directly: a more favourable label does not reliably produce a more favourable outcome, and the assumption that replacing meat with analogues is a nutritional improvement requires testing, not inference. No equivalent trial exists in any paediatric population.

### 7.5. Implications for the Diet-Quality Distinction

This body of evidence provides a concrete mechanism for the divergence between healthy and unhealthy plant-based indices. An adolescent who replaces meat with legumes, intact grains, and nuts moves along one trajectory; an adolescent who replaces meat with unfortified, high-salt analogues moves along another. The audits above establish that these two substitutions differ materially in salt, fortification, and nutrient density [48,49,50]. What they cannot show is how the difference is handled in epidemiological practice. Exposure classified by animal-food exclusion alone cannot distinguish the two trajectories, and the plant-based diet indices used throughout this review were developed precisely to overcome that limitation [11]. That not all plant foods are equally healthy is now well established [4]; that studies still routinely aggregate them is our own observation.

For adolescent clinical practice this argues against categorical advice in either direction. Analogues are neither a health food nor a hazard: they are a heterogeneous product category in which the relevant questions are whether a given product is fortified, how much salt it contains, and what it is displacing.

## 8. Psychosocial Dimensions and Disordered Eating

Adolescence is also the peak period of incidence for eating disorders, and this intersects directly with the adoption of restrictive dietary patterns. A systematic review of 20 studies including 14,391 adolescents and young adults found that the majority reported significant correlations between vegetarianism and eating disorders, while explicitly cautioning that the predominantly cross-sectional designs preclude any causal inference [58]. The direction of the association remains unresolved: a restrictive dietary pattern may be adopted as a socially acceptable vehicle for food restriction, or a genuine ethical commitment may be misclassified as pathological by screening instruments that treat meat avoidance as a symptom.

This measurement problem has prompted development of screening instruments specific to this population. The Vegetarian Vegan Eating Disorder Screener (V-EDS), an 18-item self-report tool derived from interviews with community members, clinicians, and people with lived experience, was designed explicitly to separate eating-disorder pathology from the dietary restraint inherent to vegetarian or vegan practice—for example, by probing whether a person would eat meat if survival required it, a question aimed at distinguishing ethical commitment from rigid avoidance [59]. A subsequent validation study proposed a preliminary clinical cut-off score (≥18) with good sensitivity and specificity for distinguishing clinical from non-clinical eating pathology in this population [60]. Both studies were conducted in adults; no adolescent-specific validation of the V-EDS has yet been published, so its direct applicability to this review’s target population remains to be established. In practice, screening in adolescents following plant-based diets should attend less to the presence of dietary restriction itself and more to its function: whether the pattern is flexible, socially open, and consistent with stated values, or rigid, secretive, and accompanied by weight or shape preoccupation, since it is this functional distinction—rather than the diet label—that instruments such as the V-EDS attempt to operationalise.

The clinical implication is not that plant-based eating should be discouraged, but that motivation should be assessed. Adolescents who adopt these diets for stated ethical or environmental reasons, and who maintain adequate energy intake and dietary variety, present a different clinical picture from those whose dietary change coincides with weight preoccupation, rapid narrowing of accepted foods, or falling weight-for-age. Willingness to adopt plant-based eating in adolescents is itself shaped by knowledge, skills, and perceived social norms rather than by health beliefs alone [9], which suggests that the same motivational assessment can serve both safety screening and effective counselling.

## 9. Fortification and Supplementation as Effect Modifiers

Fortification is not a secondary implementation detail; it is one of the principal determinants of whether plant-based diets in adolescence remain nutritionally safe. A 2023 paediatric systematic review explicitly recommends a variety of plant foods combined with fortification and, where needed, supplementation, in order to achieve diets that are both sustainable and nutritionally adequate [13]. A 2024 paediatric narrative review reaches the same practical conclusion, stating that vegan paediatric diets in particular require strict nutritional monitoring and adequate micronutrient supplementation [1]. The 2025 ESPGHAN Nutrition Committee position paper is more specific still, recommending that dietary intake, growth, and nutritional status be monitored regularly in vegan children, with explicit attention to protein, omega-3 fatty acids, calcium, and iron intake, and with supplementation of specific micronutrients including vitamin B12 considered essential throughout the paediatric age range [47].

Direct cohort evidence supports this position. In the Polish paediatric cohort, supplementation resolved low vitamin B12 and low 25-hydroxyvitamin D concentrations [7]. The same conclusion emerges from the systematic literature: vitamin B12 deficiency occurs in vegetarian and vegan children who do not use supplements but is rectified by supplementation [3]; blood vitamin B12 is higher in vegan children than in omnivores where supplement use is widespread [24]; and the difference in vitamin B12 concentrations between dietary groups disappears once total intake is accounted for [45]. In the Finnish study of 2- to 7-year-old children following plant-based diets, vitamin D supplement use was common (74–100% across groups) and mean 25-hydroxyvitamin D concentrations exceeded 70 nmol/L in all groups, demonstrating that vitamin D status is readily achievable within a plant-based pattern when supplementation is routine [36].

One qualification belongs here rather than in the limitations. No randomised trial has tested a structured fortification or supplementation protocol in plant-based adolescents. The recommendations above therefore rest on cohort evidence, on the demonstrated reversibility of vitamin B12 and vitamin D deficits with supplementation, and on society guidance—not on trial evidence that any particular protocol preserves growth while maintaining the cardiometabolic advantage. This is a testable gap, and Section 10.4 identifies it as a priority.

This also explains why debates about whether plant-based diets are “safe” so frequently yield conflicting answers. A well-designed plant-based diet built on fortified staples, adequate protein sources, and monitored supplementation is biologically distinct from a restrictive plant-only diet constructed around refined grains and unfortified ultra-processed replacements [4,13,50]. Treating these two patterns as a single exposure is, in our view, the principal source of inconsistency in the paediatric literature.

## 10. Discussion

The principal contribution of this review is to shift the field away from a binary comparison of vegetarian versus omnivorous diets and towards a four-part model of adolescent plant-based nutrition. No study has tested this four-part structure as an integrated framework; each component is individually supported by the literature cited, but the synthesis itself is a conceptual proposal, not an empirical finding. Its components are diet quality, for which trajectory data show opposing associations by index [5]; developmental adequacy, where cardiometabolic gain and skeletal cost can coexist in the same cohort [7]; the commercial food environment, in which most analogues are unfortified [49,50]; and implementation context, which shapes whether any of the preceding three is managed [1,47]. This model accommodates the current evidence better than exclusion-based labels alone. Healthy plant-based patterns track with more favourable metabolic outcomes in adolescents [19], whereas unhealthy patterns, ultra-processed substitutions, and poorly planned paediatric vegan diets account for much of the observed risk [7,50]—a reading consistent with the wider adult literature [4].

**Figure 1:**
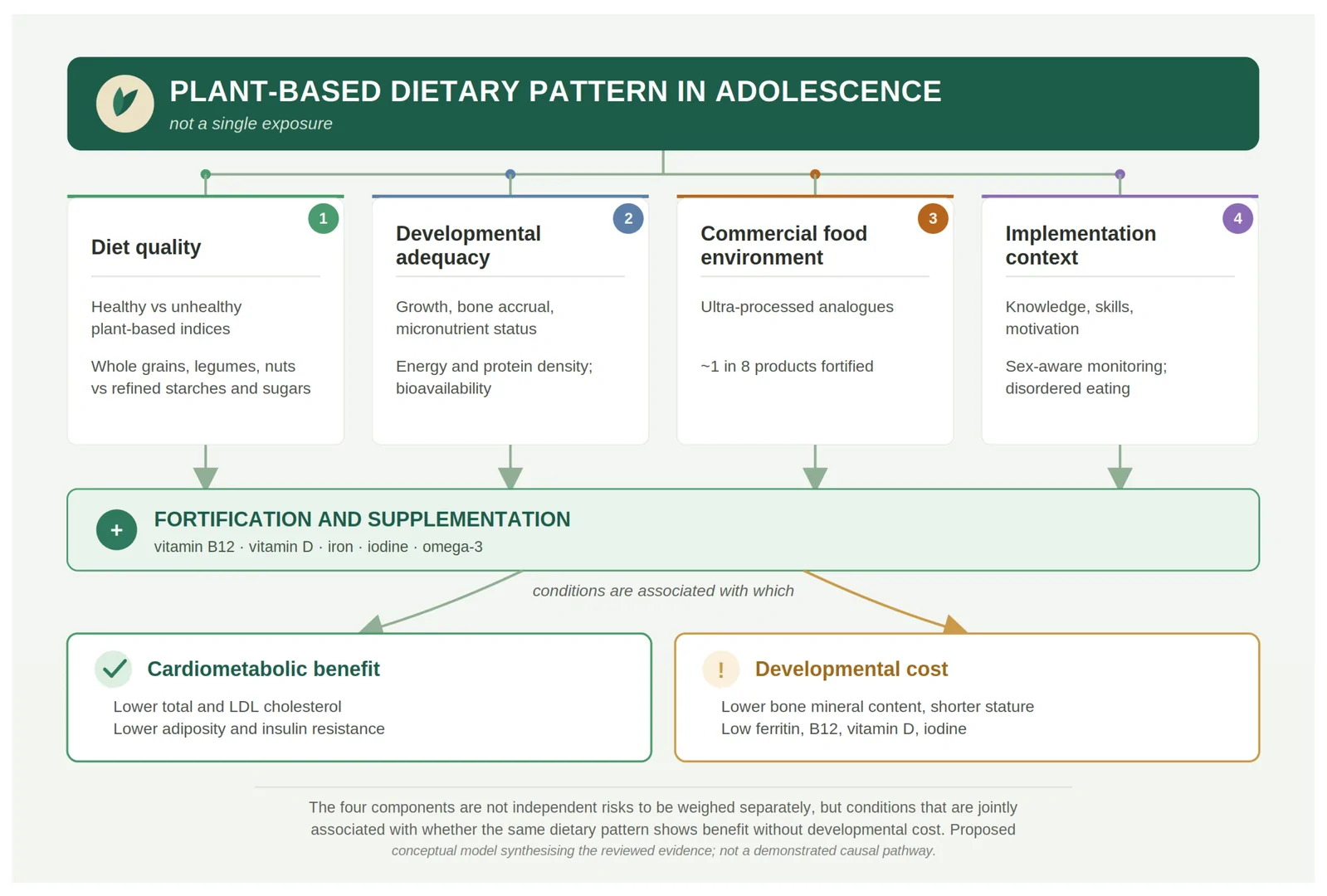
A four-part model of plant-based nutrition in adolescence. The dietary pattern is not a single exposure: four components—diet quality, developmental adequacy, the commercial food environment, and implementation context—jointly shape whether the same pattern is associated with cardiometabolic benefit without developmental cost. Fortification and supplementation act across all four as the principal effect modifier. This is a proposed conceptual model synthesising the reviewed evidence, not a demonstrated causal pathway.

Figure 1 sets out the model schematically. Its purpose is to make visible one structural claim that prose renders awkwardly: the four components do not act in parallel upon a single outcome. They act through fortification and supplementation, and the outcome then bifurcates. The same dietary pattern, passing through an adequately fortified and supplemented food environment, is associated with the cardiometabolic benefit; passing through an unfortified one, it is associated with the developmental cost. Neither branch is the intrinsic effect of eating plant-based in adolescence. Each is the effect of a set of conditions being met, or not met.

Table 1 brings together the studies that carry the main evidential weight of this review, grouped by the four components of the model, not by date of publication. Presenting them side by side makes explicit what a narrative synthesis can otherwise obscure. The model does not rest on one design, one population, or one country, but on the convergence of birth cohorts with imaging outcomes, trajectory analyses, paediatric meta-analyses, independent market audits on two continents, and a small number of randomised trials—each carrying different assumptions and different vulnerabilities to bias. It also makes visible where the evidence is thinnest. Two of the three randomised trials in the table were conducted in adults, and no row in the developmental or micronutrient sections derives from an adolescent-specific randomised design.

A recurring theme across the newer meta-analytic evidence is that the paediatric plant-based phenotype is internally consistent: lower cholesterol, lower fat mass, lower height and bone mineral content, and lower iron and vitamin B12 status all co-occur [3,23,24]. We propose—as a hypothesis rather than an established finding—that these are not independent outcomes to be weighed separately but manifestations of a single underlying pattern of lower energy and protein density combined with lower bioavailability of specific micronutrients. If correct, interventions that address the underlying pattern (adequate energy, adequate protein quality, fortified staples) should move several outcomes simultaneously. This prediction is testable and, to our knowledge, has not been tested.

### 10.1. The Case for Sex-Specific Monitoring

Evidence from a single adolescent cohort suggests sex differences in cardiometabolic associations, with unhealthy plant-based trajectories more strongly associated with adverse anthropometric and insulin-related outcomes in males, and healthy trajectories more strongly associated with favourable outcomes in females [5]. In parallel, paediatric nutrient reviews and meta-analyses identify adolescent girls as a group at greater risk of iron deficiency and anaemia [13,23]. Taken together, these observations convert a generic safety discussion into an adolescence-specific and sex-aware one, and may support differentiated monitoring rather than uniform screening, although this recommendation remains preliminary: the sex-specific findings derive from a single cohort and require replication before they can inform practice.

### 10.2. Implementation Rather than Ideology

Adolescents frequently lack the knowledge, culinary skills, and motivation required to adopt healthier plant-based eating, and education that builds familiarity and practical skills is repeatedly identified as a practical lever [9]. Framing the clinical conversation with adolescent patients around food quality, fortified staples, and adequate protein and micronutrient sources is likely to be more productive than framing it around the legitimacy of the dietary choice itself. The heterogeneity of national recommendations [3] means that clinicians frequently cannot defer to a single authoritative guideline, which increases the value of a consistent, mechanism-based counselling framework anchored in international society guidance [47].

### 10.3. Limitations

Several limitations should be acknowledged. The adolescent-specific evidence base remains small, and much of the most informative data on growth and bone outcomes derives from younger children, not from adolescents [7,36]. Exposure definitions are heterogeneous, dietary intake is largely self-reported, and residual confounding by socioeconomic status, physical activity, and overall lifestyle cannot be excluded in observational designs. The certainty of evidence in the available paediatric meta-analyses has been rated as low or very low, with a substantial proportion of included studies at high risk of bias, and nearly all primary studies are cross-sectional [24,37]. Randomised evidence in adolescents is essentially absent; the identical-twins trial that provides the strongest causal signal in this field was conducted in adults [25].

The composition of the evidence base deserves to be quantified, not implied. Of the 60 references cited, 23 (38%) derive from adult populations, 24 (40%) include adolescents, seven are audits of product composition, four are paediatric studies conducted below the age of adolescence, one pools children with adults, and one is qualitative. A substantial part of the adult-derived evidence supports caution rather than benefit—fracture risk, bone turnover, lower ferritin, reduced iron bioavailability, and a null substitution trial—so extrapolation from adults in this review tempers the claims made for plant-based diets in adolescence at least as often as it strengthens them. It is worth stating plainly which of this review’s conclusions do not rest on adolescent data. Four do not. The fracture consequences of strict exclusion come entirely from adult cohorts and meta-analyses [31,32,33]. The bone-turnover mechanism linking low protein and calcium intake to raised parathyroid hormone is documented in adults [34,42] and in children aged 2–7 years [36], but not in adolescents. The iron bioavailability figures that justify a higher intake requirement are adult isotope estimates [40]. And the only trial of substituting meat with analogues was conducted in adults at elevated diabetes risk [57]. Conversely, four conclusions do rest on adolescent-inclusive data: the diet-quality gradient for cardiometabolic outcomes [5,6,19]; the leaner phenotype with lower bone mineral content [23,24]; the vitamin B12 and zinc vulnerabilities [44,45]; and the association between ultra-processed food intake and cardiometabolic health [55]. Readers should weight the two groups differently. Market audits characterise product composition rather than actual adolescent intake, so their translation into exposure remains inferential [48,50]. Long-term adherence and attrition patterns in plant-based adolescent cohorts remain incompletely documented.

### 10.4. Directions for Future Research

Four priorities follow from the gaps identified above. First, longitudinal studies pairing cardiometabolic endpoints with growth, pubertal, and bone outcomes in the same cohort. Second, explicit quantification of ultra-processed plant food intake as an exposure distinct from plant-food intake. Third, systematic reporting of iodine, omega-3 fatty acid, and protein-quality data, which remain sparse. Fourth, and most important, intervention trials testing whether structured fortification and supplementation protocols preserve the cardiometabolic advantage while removing the micronutrient and skeletal penalties. Harmonisation of national paediatric recommendations would also reduce the practical uncertainty faced by clinicians [3,47].

## 11. Conclusions

Healthy plant-based diets in adolescence appear cardiometabolically favourable, and lower total and LDL cholesterol is their most consistently replicated effect, although HDL cholesterol falls alongside it. Their safety and durability, however, depend on four conditions: a predominance of minimally processed foods rather than unfortified ultra-processed analogues; adequate energy and protein quality to protect growth and bone accrual; systematic attention to vitamin B12, vitamin D, iron, iodine, and omega-3 fatty acids. These nutrient do not all require the same approach: vitamin B12 supplementation is routinely indicated once animal-source foods are excluded, vitamin D, iodine, and omega-3 intake are substantially addressed through fortified staples and targeted supplementation, and iron warrants regular monitoring of status with supplementation individualised to documented deficiency rather than applied universally; and age- and sex-aware implementation that includes assessment of dietary motivation. The relevant clinical question is therefore not whether adolescents should eat plant-based diets, but how such diets should be constructed and monitored so that cardiometabolic gain is not purchased at the cost of growth, bone accrual, or micronutrient sufficiency.

## Figures and Tables

**Table 1:** Key studies informing the four-part model of plant-based nutrition in adolescence.

Study [Ref]	Design	Population	Evidence Base	Main Finding as Reported in the Source
**Metabolic**				
Murcia-Lesmes [6]	Prospective cohort, 4 y (SI! Programme)	886 Spanish adolescents, 12 y at entry	Adolescent	High vs. low PHDI adherence (Q4 vs. Q1): HR 0.19 (0.11–0.34) for new-onset high blood pressure; 0.34 (0.18–0.65) for raised triglycerides; 0.26 (0.13–0.50) for raised non-HDL cholesterol.
Marchese [5]	Group-based trajectory modelling, 14→28 y (Raine Study)	417 Australian participants	Adolescent → adult	Females, higher hPDI: insulin β −1.11 (−2.12, −0.09); HOMA-IR −0.25 (−0.48, −0.01); systolic BP −2.75 (−5.31, −0.19); hs-CRP −1.53 (−2.82, −0.23). Males, higher uPDI: waist +3.12 cm (0.61, 5.63); HOMA-IR +0.35 (0.07, 0.63).
Mokhtari [19]	Cross-sectional	203 Iranian adolescents with overweight or obesity, 12–18 y	Adolescent	hPDI tertile 3 vs. 1: 84–85% lower odds of metabolically unhealthy obesity. uPDI tertile 3 vs. 1: OR 3.95 (1.41–11.12) and 4.06 (1.31–12.57). Associations stronger in girls.
Landry [25]	8-week randomised controlled trial, identical twins	22 twin pairs (*n* = 44), mean age 39.6 y	Adult	Vegan vs. omnivorous diet: LDL cholesterol −13.9 mg/dL (−25.3, −2.4); fasting insulin −2.9 μIU/mL (−5.3, −0.4); body weight −1.9 kg (−3.3, −0.6).
**Bone/growth**				
Desmond [7]	Cross-sectional with DXA and deuterium dilution	187 Polish children 5–10 y (63 vegetarian, 52 vegan, 72 omnivore)	Paediatric, 5–10 y	Vegans: LDL −24 mg/dL (−35.2, −12.9); serum B12 −217.6 pmol/L (−305.7, −129.5); shorter stature. Bone mineral content deficit persisted after body-size adjustment in vegans (total body minus head −3.7%; lumbar spine −5.6%) but attenuated in vegetarians.
Lotti [23]	Systematic review and meta-analysis	59 studies, 48,626 participants <18 y (7280 lacto-ovo vegetarian, 1289 vegan, 40,059 omnivore)	Paediatric, <18 y	Leaner phenotype: lower height, weight, BMI z-score, fat mass and bone mineral content in lacto-ovo vegetarians; shorter stature in vegans. Lower ferritin and 25(OH)D; increased odds of iron deficiency and anaemia. Group means for most nutrients remained within paediatric reference ranges.
Koller [24]	Systematic review and meta-analyses	18 studies (17 cross-sectional, 1 RCT), 0–18 y	Paediatric, 0–18 y	Lower protein, calcium and riboflavin intake; lower ferritin, HDL, LDL and height in vegans. Blood vitamin B12 higher owing to supplement use. Certainty of evidence low or very low; 7 of 18 studies at high or very high risk of bias.
Sun [20]	Prospective cohort, diet at 8 y, outcomes at 10 and 13 y (Generation R)	3340 Dutch children, 8–13 y	Paediatric, 8–13 y	Higher hPDI: fat-free mass index z +0.05 (0.01, 0.10); body fat percentage z −0.05 (−0.09, −0.01). Higher uPDI: fat-free mass index −0.08 (−0.12, −0.03), with lower height and weight. All three indices associated with lower vitamin B2, B12 and calcium intake.
Movassagh [30]	Longitudinal cohort with DXA, adolescence to young adulthood (PBMAS)	125 adolescents (115 at adult follow-up, mean 28.2 y)	Adolescent → adult	“Vegetarian-style” pattern, quartile 3 vs. 1: young-adult total-body bone mineral content +8.5%; femoral neck BMC +10.6%. Dietary pattern scores tracked moderately (0.47–0.63). Pattern included eggs, fruit juice and low-fat milk.
Itkonen [36]	Cross-sectional, bone and mineral metabolism	71 Finnish children 2–7 y (29 vegan, 18 vegetarian, 24 omnivore)	Paediatric, 2–7 y	Linear trend towards higher parathyroid hormone in plant-based groups despite adequate vitamin D status. Vitamin D supplement use, 74–100%; mean 25(OH)D >70 nmol/L in all groups. Protein intake 12–14 E% vs. 16–17 E% in omnivores.
Tong [31]	Prospective cohort, mean 17.6 y follow-up (EPIC-Oxford)	54,898 participants (1982 vegans at baseline)	Adult	Vegans vs. meat eaters, adjusted for BMI: hip fracture HR 2.31 (1.66–3.22); total fracture 1.43 (1.20–1.70). Vegetarians: hip fracture 1.25 (1.04–1.50). No paediatric equivalent exists.
**Micronutrients**				
Neufingerl [13]	Systematic review	30 studies, children and adolescents 2–18 y	Paediatric, 2–18 y	All dietary groups showed risk of inadequate vitamin D and calcium intake. Plant-based diets specifically more likely to show inadequate vitamin B12, iron and zinc.
Jensen [45]	Systematic review and meta-analysis	Children and adolescents 5–18 y on plant-based diets	Paediatric, 5–18 y	Vitamin B12 −97 pmol/L (−187, −7) vs. omnivores. Effect significant for vegan and macrobiotic but not vegetarian diets, and nullified when total vitamin B12 intake was accounted for.
**Food environment**				
Alessandrini [48]	Cross-sectional market audit	207 plant-based and 226 meat products, 14 UK retailers	Product data	Lower energy density, total and saturated fat, and protein; higher fibre. Salt significantly higher in 5 of 6 categories; ~75% did not meet UK salt targets. 14% vs. 40% classified “less healthy” by the UK nutrient profiling model.
Curtain [52]	Cross-sectional market audit	137 products, 4 Australian supermarkets, 2019	Product data	Fortified with vitamin B12 in 24%, iron in 20%, zinc in 18%. Only 4% low in sodium (range 58–1200 mg/100 g).
Melville [50]	Cross-sectional market audit	790 products, Australia	Product data	Higher Health Star Rating, lower saturated fat and sodium, higher total sugar than meat. Exactly 84% of analogues and 89% of meat products ultra-processed. Only 12.1% fortified with iron, vitamin B12 and zinc.
Brodersen [49]	Cross-sectional market audit	298 analogues vs. 294 animal-based products, 10 German chains (>70% of market), 2024	Product data	Nutri-Score more favourable in 6 of 9 subcategories; salt higher in 3 of 4 meat-analogue categories (red meat 1.8 vs. 0.6 g/100 g). Only 12% fortified with any micronutrient; no product contained added vitamin D or iodine.
Nicol [46]	Cross-sectional market audit with substitution modelling	300 plant-based dairy and fish alternatives, UK retail, 2020	Product data	Iodine fortification in 28% of milk and 6% of yoghurt alternatives, vs. calcium fortification in 88% and 73%. No cheese or fish alternative iodine fortified. Substituting 3 daily dairy portions with unfortified alternatives: iodine −97.9% (124 vs. 2.6 μg).
Drewnowski [51]	Database analysis of branded products	118 plant-based meat alternatives vs. 450 raw meat entries, USA	Product data	Nutrient density (NRF5.3) significantly lower for alternatives. Mean sodium 660 vs. 60 mg/100 g for beef; mean protein 17 vs. 20 g/100 g. Fortification with B vitamins, vitamin D, iodine, zinc and omega-3 minimal.
Toh [57]	8-week randomised controlled trial, substitution design	89 participants at elevated diabetes risk, Singapore	Adult	Substituting 2.5 daily servings of meat with analogues produced no difference in LDL cholesterol (primary outcome). Diastolic BP lower with analogues, but glucose time in range better with animal meat (94.1% vs. 86.5%).
**Psychosocial**				
Sergentanis [58]	Systematic review	20 studies, 14,391 adolescents and young adults	Adolescent–young adult	Most studies reported significant correlations between vegetarianism and eating disorders; predominantly cross-sectional designs preclude causal inference.

The evidence base column classifies each study by the population from which its finding derives. Studies marked “Adult” are included because no paediatric or adolescent equivalent exists; they are cited for mechanistic or methodological rather than directly transferable evidence. “Product data” denotes audits of commercially available foods, which describe composition rather than intake or health outcome. Across the full reference list, 23 of 60 sources (38%) are adult-derived; Section 10.3 identifies which conclusions rest on them. Abbreviations: BMC, bone mineral content; BMI, body mass index; BP, blood pressure; DXA, dual-energy X-ray absorptiometry; E%, percentage of total energy; HOMA-IR, homeostatic model assessment of insulin resistance; hPDI, healthful plant-based diet index; HR, hazard ratio; hs-CRP, high-sensitivity C-reactive protein; OR, odds ratio; PBMAS, Saskatchewan Paediatric Bone Mineral Accrual Study; PHDI, Planetary Health Diet Index; uPDI, unhealthful plant-based diet index; 25(OH)D, 25-hydroxyvitamin D.

## Data Availability

No new data were created or analysed in this study. Data sharing is not applicable to this article.

## Abbreviations

The following abbreviations are used in this manuscript:

BMC	bone mineral content
BMD	bone mineral density
BMI	body mass index
CI	confidence interval
ESPGHAN	European Society for Paediatric Gastroenterology, Hepatology and Nutrition
HDL	high-density lipoprotein
HOMA-IR	homeostatic model assessment of insulin resistance
hs-CRP	high-sensitivity C-reactive protein
LDL	low-density lipoprotein
PHDI	Planetary Health Diet Index
PTH	parathyroid hormone
25(OH)D	25-hydroxyvitamin D
